# Editorial Notes

**Published:** 1901-12

**Authors:** 


### Editorial Notes.

**University College: Faculty of Medicine.**—For many years past the custom of an opening introductory lecture at the commencement of the medical session in the Bristol Medical School has been discontinued: the distribution of prizes has been the only public function, and even this had fallen into abeyance for the last five or six years. The Medical Faculty of the University College may this year be congratulated on their success in having obtained the services of **Sir Frederick Treves, K.C.V.O., C.B., F.R.C.S.**, to distribute the prizes and address the students on October 25th, 1901, in the large hall of the College, which was crowded by an enthusiastic audience, consisting of the College staff, the students, and their friends.

From the report of Dr. Markham Skerritt (the Dean of the Faculty) we learn that the Faculty of Medicine have much pleasure in recording the thoroughly satisfactory condition of their department, and the good work that has been accomplished during the past year, which is attested by the success of their students at the various examinations of the qualifying bodies. They have been much gratified at the excellent working of the arrangement whereby the Bristol Royal Infirmary and the Bristol General Hospital are amalgamated for clinical teaching. As the Bristol Royal Hospital for sick children and women and the Bristol Eye Hospital are also to be opened to their students, the total number of beds thus available for teaching is 602. Few schools of medicine offer to their students so wide and varied a field.

In the course of an exceptionally interesting and learned address, Sir Frederick Treves declared with truth that the medical man of to-day had become an actual factor in the state, and dealt with great questions, controlled enormous issues, and became weighted with great responsibilities. He characterised the profession of medicine as the "profession of romance." Was it realised that there was much more romance in the new worlds discovered in their profession than in the days of the old princely adventures that took place from the port of Bristol when Giovanni Cabotto sailed from Bristol? Could one imagine the feeling of the man who would one day discover a cure for cancer? For it would be discovered. In conclusion, Sir Frederick remarked that a man must not follow the profession of medicine merely for the money it might bring into his pocket. In the numerous acts of unselfishness the profession displayed, in the pain it alleviated, and in the gratitude it earned, the medical man was one of the very few who could say: "I may not have gold of this coinage or of that, but I know something of the wealth which, in a very proper sense, is beyond the dream of avarice."

In the evening Dr. Skerritt presided at the Medical School dinner, held at the Royal Hotel, at which Sir Frederick Treves was the guest of the evening. Dr. Skerritt was well supported by the Right Hon. the Lord Mayor of Bristol and a large number of Bristol medical men and old and present medical students.

The **New Bacteriological Department** at the Bristol Royal Infirmary was opened by Sir Frederick Treves in the afternoon. In 1897, on the recommendation of the Faculty, an honorary bacteriologist had been appointed. His work was carried on under considerable disadvantage, owing to the inadequacy of accommodation provided for his department. In 1901 certain rooms near the Museum became available, and by some architectural ingenuity four convenient laboratories for bacteriological and pathological purposes have been provided. These rooms are lighted by electricity and well equipped with the most modern apparatus for bacteriological work. Sir Frederick Treves stated that "to establish a laboratory of the character of that of which Professor Stanley Kent would be hon. bacteriologist involved a considerable outlay of money. The question as to its need was answered by the fact that bacteriology stood at the very van of medical and surgical progress. Those who were interested in the charitable side of the hospital work must have some satisfaction in feeling that there was no handsomer present, no more suitable gift to any institution of that character, than a thoroughly well-equipped bacteriological laboratory." The old bacteriological department has done very useful work for some years, but the work had quite outgrown its limited accommodation. Now, however, this work can flourish and expand, and prove a great source of strength to the clinical work of the Royal Infirmary, under our very able colleague who has charge of it.

In connection with the opening of the new bacteriological department, it will be interesting to recall the various **alterations that have recently been made at the Royal Infirmary**; and we have given a brief *résumé* of these under the head of "Local Notes," p. 379, from which it will be seen, in spite of the difficulties associated with the alterations of an old building, and the absolute limitations of space at their disposal, the Committee of the Bristol Royal Infirmary have not been slow to institute such amendments and extensions as have been necessary to maintain the prestige of one of the oldest hospitals in the country, in which much good work has been accomplished for a period of over 160 years. It is worthy of note that these alterations and improvements have necessitated an expenditure of about twenty-five thousand pounds.

At the *Annual Meeting of the University College, Bristol*, the Lord Bishop of Hereford stated that the report showed conclusively that the College was sending its roots deeper and deeper into the life of the city, and no one who had heard the report read, especially those pages dealing with the original work of professors, lecturers, and former students, could help feeling what a valuable addition to the intellectual life of the city was within the walls of the College. The Bishop, referring to the more general question of education and its condition in England just now, said they were reminded on all sides that one of the greatest calls upon Englishmen was to improve their higher education, both technical and general, and they noticed how in other places this call was producing very remarkable results. They had all observed how it had produced quite lately the new University of Birmingham, and those who had studied the question again observed what remarkable effect the establishment of this new University had upon the northern part of England, how it seemed likely to cause the development of three or four independent Universities. It looked as if before long there would be Universities for Manchester, Liverpool, and probably Yorkshire, and possibly one or two others added to the present list. In the face of this he would ask, Why not Bristol and West of England University also?

He hoped this question was being seriously considered by a great many of the citizens of Bristol, because if there was any city in England which, from its history, dignity, its great place in the life of the country, deserved to be the site for a University serving a wide area, he ventured to think that it was the city of Bristol. He looked forward, and hoped for the time with interest, to their having the Bristol University College and the Merchants' College as the first constituent colleges of the University of Bristol—"the lantern of the West" they would call it,—when the city would be the University home of the wide West of England.

By the death of **Sir William MacCormac, Bart., K.C.V.O.,** the Medical Profession loses one of its most distinguished members. The nation will not soon forget that by his offering his services to the British Government in connection with the South African War, he rendered most valuable help in the organisation of the medical and surgical work at the chief centres of the campaign. And there can be little doubt that the strain and anxiety involved in this patriotic work undermined his health and precipitated his death. In Bristol he will be remembered with gratitude for the kind and practical interest he displayed towards our oldest hospital, in coming down to open the new operating theatre in memory of Mr. Greig Smith.

The public **Opening of the Newport and Monmouthshire New Hospital** by the Right Hon. Lord Tredegar, the Lord-Lieutenant of Monmouthshire, on August 5th, 1901, was signalised by a great demonstration of the Friendly Societies and trade associations of the district at Newport. These assembled at the Town Hall, and formed a large and representative procession, which paraded the principal streets of the town and then assembled in front of the new building, where the opening ceremony took place. The site for the hospital had been generously presented by Lord Tredegar, and one of the medical staff (Dr. Garrod Thomas) had shown his belief in the necessity for such a hospital by contributing the sum of £5,000 towards the cost; this in fact gave the start to the whole movement. The medical and surgical staff of the hospital must feel that their appeal for increased and improved hospital accommodation in Newport has met with a generous response from the numerous subscribers, who will no doubt be urged to continue their philanthropic work by providing funds for the continued maintenance of the hospital on a most liberal scale. Further details of the hospital, together with an illustration of the frontage, will be found under Local Notes, p. 380.

The fourth resolution adopted by the **British Congress on Tuberculosis** is as follows:—"That the provision of sanatoria is an indispensable part of the measures necessary for the diminution of tuberculosis." This provision is rendered all the more necessary from the fact that all our general hospitals have a rule by which cases of advanced consumption are considered to be inadmissible. It is true that this rule has been frequently allowed to fall into abeyance in the past, but in accordance with the modern views relating to the contagious character of the disease this rule is now being much more rigidly enforced; accordingly no hospitals are now available for consumptives, and special hospitals for them become essential both for those in the early and in the late stages.

There are eighty-three public sanatoria in Germany, sufficient to accommodate 12,000 patients annually. In England a few such sanatoria have been erected, but we are altogether behind both the Continent and the United States in this respect. One of the most complete sanatoria yet erected is that at Pinewood, about five miles from Woking. It has been recently erected at a cost of over £20,000 by Mr. Lionel Phillips and Mr. Rübe, under the charge of the National Association for the Prevention of Consumption. In our own district it is contemplated that the proposed sanatorium at Winsley will provide some accommodation for the counties of Bristol, Gloucester, Somerset and Wilts. The site has been purchased, the ground has been levelled, and the executive are only waiting some augmentation of the funds before entering into a contract for the building. The city of Bristol should be eager to do its share in the good work of curing, preventing, and stamping out tubercular disease from the world.

For the following notes on **plague and diphtheria** we are indebted to our Medical Officer of Health, Dr. David S. Davies, and we would wish to direct attention to his paper on "The Policy of Rat-free Ships" which was recently read at the annual meeting of the Association of Port Sanitary Authorities[1]

Another paper by Dr. Davies, on "Plague and its Prevention as a Disease Communicable from Animals to Man," was published in the *British Medical Journal*.[2]

The fresh introduction (or is it recrudescence?) of plague into Glasgow, and the undoubted implication of rats, together with the appearance of the disease in Liverpool (whence, sea-borne or from Glasgow?) directs attention to its epizoötic nature, and prompts direct enquiry into the sufficiency of the methods at present directed against its importation. In the Bristol Port Report for 1900, the Medical Officers direct attention to this point, and argue with some force that measures designed

[1] *Vide Western Daily Press*, Oct. 30, 1901.
[2] 1901, ii. 337.

against a purely human disease, as are those of the Cholera, Plague, and Yellow Fever Order of 1896, while providing safeguards against the introduction of the disease by human agency, are impotent against the more subtle but proved possibility of its introduction by infected rats. This defect in the Order is imperfectly remedied by a Memorandum issued in April of this year, wherein the Local Government Board recommend, but do not provide, compulsory powers for certain measures designed to prevent the communication of ship rats, if infected, with shore rats; this Memorandum practically approves the steps which many alert Port Authorities had previously adopted without official sanction. In January, actual experience in Bristol had already demonstrated the wisdom of such precautions, and a ship from Smyrna, with many plague-killed rats on board, had been so handled as to prevent the transmission of infection ashore. The ship was overrun with rats, as, in addition to the 13 dead of plague on board, 213 others were subsequently destroyed by fumigation. The crew was, in this instance, absolutely unaffected; and this may readily occur, as the crew sails the ship, and does not necessarily come into relation with the work in the holds, into which, especially in grain boats, rats may readily be introduced from infected granaries. The danger is obviously to the labourers discharging infected holds. At the Cheltenham meeting of the British Medical Association, the Bristol Port Medical Officer followed up the subject. If the urgency of rat infection is admitted, and if Australia has succeeded in securing a service of rat-free merchant ships, why should England, or Europe, wait for the goad of disaster before adopting a similar simple and wise policy? At present, droves of rats, useless and annoying at all times, are permitted to be carried from port to port, multiplying the chances of infection manyfold. If this risk were removed, the risk of serious plague infection in Europe would be practically annihilated, for human introduction can readily be handled, as shown at Glasgow and Hull; whereas rat introduction, as demonstrated in Sydney, is no less than a calamity. And yet, so slowly does national intelligence move, a town with a single bubonic case of plague would be scheduled "infected" by the European powers, whereas the reported invasion by rats from an infected ship would probably escape serious attention. The question is international, as was the question of cholera, discussed at the Venice Convention. The new conditions of epizoötic implication need fresh international discussion towards a policy of rat-free ships, lest the blamelessness allowed by Koch to governments who are satisfied with regulations conceived before enlightenment be swallowed up in the sin of neglected opportunity.

The article on Plague in the *Quarterly Review* for October, 1901, gives a concise, graphic and most interesting history of the disease, up to the first Glasgow invasion, but, curiously enough, omitting the Hull pneumonic outbreak. The writer, however, while admitting the importance of the danger averted in January in Bristol by the anticipatory precautions adopted against ship-borne rat-infection, and while recognising the epizoötic character of plague in general as the key to the situation, fails in the end to grasp the fact that the influence of "insanitary" conditions in spreading plague is chiefly or altogether a measure of the opportunities afforded by such conditions for rat-infection. This conclusion may, at any rate, be predicated for Western populations.

Diphtheria has not for many years been a source of great mortality or of special anxiety in Bristol. The mortality per 100,000 living for the ten years 1890-99 is shown by the Registrar-General to have been only 14, compared with a mortality rate for the 33 large towns of 33 per 100,000. In the year 1899 it was only 10, while in 1900 it rose to 31. Bristol was at length sharing in the Western recrudescence of this disease. But great as was the increase observed, other towns suffered more in proportion: thus, the Cardiff figures for 1899—1900 were 33 and 42, and the Swansea figures 136 and 58. The rise in Bristol, as in other towns, is independent of the "fever" rate, and bears no constant relation to the sanitary condition of affected towns; thus, while the fever rate of Leicester is only 18, over 10 years, the diphtheria rate rose in 1899 and 1900 to 106 and 151. The Medical Officer of Health lays great stress upon the importance of unsuspected nasal diphtheria in "carrying" the disease. This "carrier" form, which, he points out, may exist without any constitutional symptoms, so that the patient attends school and mixes freely with other children in the street or playground, is frequently a source of fresh outbreaks, and accounts for much of the difficulty in controlling this troublesome disease.

It is now commonly understood that milk can be deprived of danger from all kinds of germs by raising it to the temperature known to be fatal to these germs. Less than the boiling temperature suffices for this purpose, and hence the custom is growing of submitting the milk to a process of what is called pasteurisation instead of complete boiling. This, however, has proved itself to be beset with fallacies, inasmuch as many cases of scarlet fever have recently been traced to milk which was called pasteurised. It behoves those who undertake to provide germ-free or sterilised milk to adopt every guarantee that their process is efficient; it is little short of a scandal that the victims of this abuse do not appear to have any legal remedy.

